# Schizophrenia Identification Using Multi-View Graph Measures of Functional Brain Networks

**DOI:** 10.3389/fbioe.2019.00479

**Published:** 2020-01-15

**Authors:** Yizhen Xiang, Jianxin Wang, Guanxin Tan, Fang-Xiang Wu, Jin Liu

**Affiliations:** ^1^School of Computer Science and Engineering, Central South University, Changsha, China; ^2^Hunan Provincial Key Lab on Bioinformatics, Central South University, Changsha, China; ^3^Division of Biomedical Engineering and Department of Mechanical Engineering, University of Saskatchewan, Saskatoon, SK, Canada

**Keywords:** Schizophrenia identification, fMRI, functional brain networks, multi-view graph measures, SVM

## Abstract

Schizophrenia (SZ) is a functional mental disorder that seriously affects the social life of patients. Therefore, accurate diagnosis of SZ has raised extensive attention of researchers. At present, study of brain network based on resting-state functional magnetic resonance imaging (rs-fMRI) has provided promising results for SZ identification by studying functional network alteration. However, previous studies based on brain network analysis are not very effective for SZ identification. Therefore, we propose an improved SZ identification method using multi-view graph measures of functional brain networks. Firstly, we construct an individual functional connectivity network based on Brainnetome atlas for each subject. Then, multi-view graph measures are calculated by the brain network analysis method as feature representations. Next, in order to consider the relationships between measures within the same brain region in feature selection, multi-view measures are grouped according to the corresponding regions and Sparse Group Lasso is applied to identify discriminative features based on this feature grouping structure. Finally, a support vector machine (SVM) classifier is employed to perform SZ identification task. To evaluate our proposed method, computational experiments are conducted on 145 subjects (71 schizophrenic patients and 74 healthy controls) using a leave-one-out cross-validation (LOOCV) scheme. The results show that our proposed method can obtain an accuracy of 93.10% for SZ identification. By comparison, our method is more effective for SZ identification than some existing methods.

## 1. Introduction

Schizophrenia (SZ) is a functional mental disorder which caused by genetic factors and environmental effects. Patients with SZ (SZs) share some common symptoms which include depression, hallucinations, cognitive dysfunction and disorganized thinking (Marín, 2012). Impairments of this disorder cover multiple cognitive areas, including memory (He et al., 2012), attention and executive function (Heinrichs and Zakzanis, 1998). One percent of the population is affected by the serious psychiatric disease worldwide (Ripke et al., 2013). The clinical diagnosis of SZ relies mainly on mental state examination rather than any biomarker (Arbabshirani et al., 2013; Liu et al., 2017d) since the cause and mechanism of the disease are not clearly revealed. However, this diagnosis method is usually subjective and not completely effective. Therefore, it is urgent to find an objective method to realize the automatic diagnosis of SZ and improve the accuracy of recognition.

Nowadays, Magnetic resonance imaging technology has been widely used in various studies related to brain disease diagnosis (Nieuwenhuis et al., 2012; Liu et al., 2016, 2017b,c, 2018a; Yang and Wang, 2018). Since SZ is reported to be a functional disease, functional magnetic resonance imaging (fMRI) is increasingly used to study brain dysfunction in patients with mental illness (Castro et al., 2011; Huang et al., 2018; Liu et al., 2018b; Moghimi et al., 2018; Chen et al., 2019). In addition, fMRI provides a database for functional analysis of these brain diseases owing to it's massive spatial and temporal information.

In recent years, the number of neurobiological literatures using fMRI to study SZ disease has increased significantly. fMRI is usually applied to discover anomalous patterns present in activation maps [i.e., Regional Homogeneity (REHO), Amplitude of Low Frequency Fluctuations (ALFF), fractional Amplitude of Low Frequency Fluctuations (FALFF)] (Guo et al., 2014; Chyzhyk et al., 2015; Huang et al., 2018) of SZ. These activation maps are widely used as potential clinical biomarkers for the diagnosis of SZ. For example, Huang et al. (2018) used tree-guided group sparse learning method to perform feature selection on fALFF data in multi-frequency bands, and then used multi-kernel learning (MKL) method to achieve an accuracy of 91.10% on 34 subjects. Chyzhyk et al. (2015) combined these activation maps by using extreme learning machines and successfully distinguished SZs from healthy controls (HCs). However, these methods focus on the voxel-wise information in these maps rather than the connectivity between regions of interest (ROIs).

Functional connectivity has been reported to analyze the differences in the functional organization of brain networks between patients and HCs (Lynall et al., 2010; Pettersson-Yeo et al., 2011). Functional connectivity networks are usually derived from fMRI data (Van Den Heuvel and Pol, 2010; Craddock et al., 2013). Nodes of a functional brain network could be the voxels of fMRI data, ROIs defined by brain atlas or the discrete regions with similar size by randomly parcellating the brain (Fornito et al., 2013). Links of a functional brain network could be determined by the correlations estimated from time courses between pairs of nodes (Liu et al., 2017a). For example, Yu et al. (2015) created functional brain network using group ICA and Pearson correlation coefficient, and they found the new evidence about altered dynamic brain graphs in SZ. Abraham et al. (2017) investigated the most predictive biomarkers for Autism spectrum disorders (ASD) by building participant-specific connectomes from functionally-defined brain areas. For these methods, the connections between all pairs of nodes in a brain network are employed as features, but the topological measures of connectivity networks are not considered.

To quantitatively analyze functional brain networks, graph theoretical analysis is employed for investigating the topological organization of functional connectivity (Anderson and Cohen, 2013; Brier et al., 2014). The most commonly used graph measures include betweenness centrality, degree, local efficiency, participation coefficient, average clustering coefficient, average path length, global efficiency, and small-worldness (Liu et al., 2017a). These topological measures have been applied in the brain disease classifications (Cheng et al., 2015; Khazaee et al., 2015, 2017; Moghimi et al., 2018). For example, Moghimi et al. (2018) calculated a set of 25 graph measures including global and local measures for each subject and obtained a classification accuracy of 80% with a double-cross validation scheme. Cheng et al. (2015) achieved an accuracy of 79% by using betweenness centrality measure in SZ identification, and they found that changes in functional hubs were associated with SZ. Overall, these methods using graph measures for SZ identification have not achieved a good classification performance.

**Figure 1:**
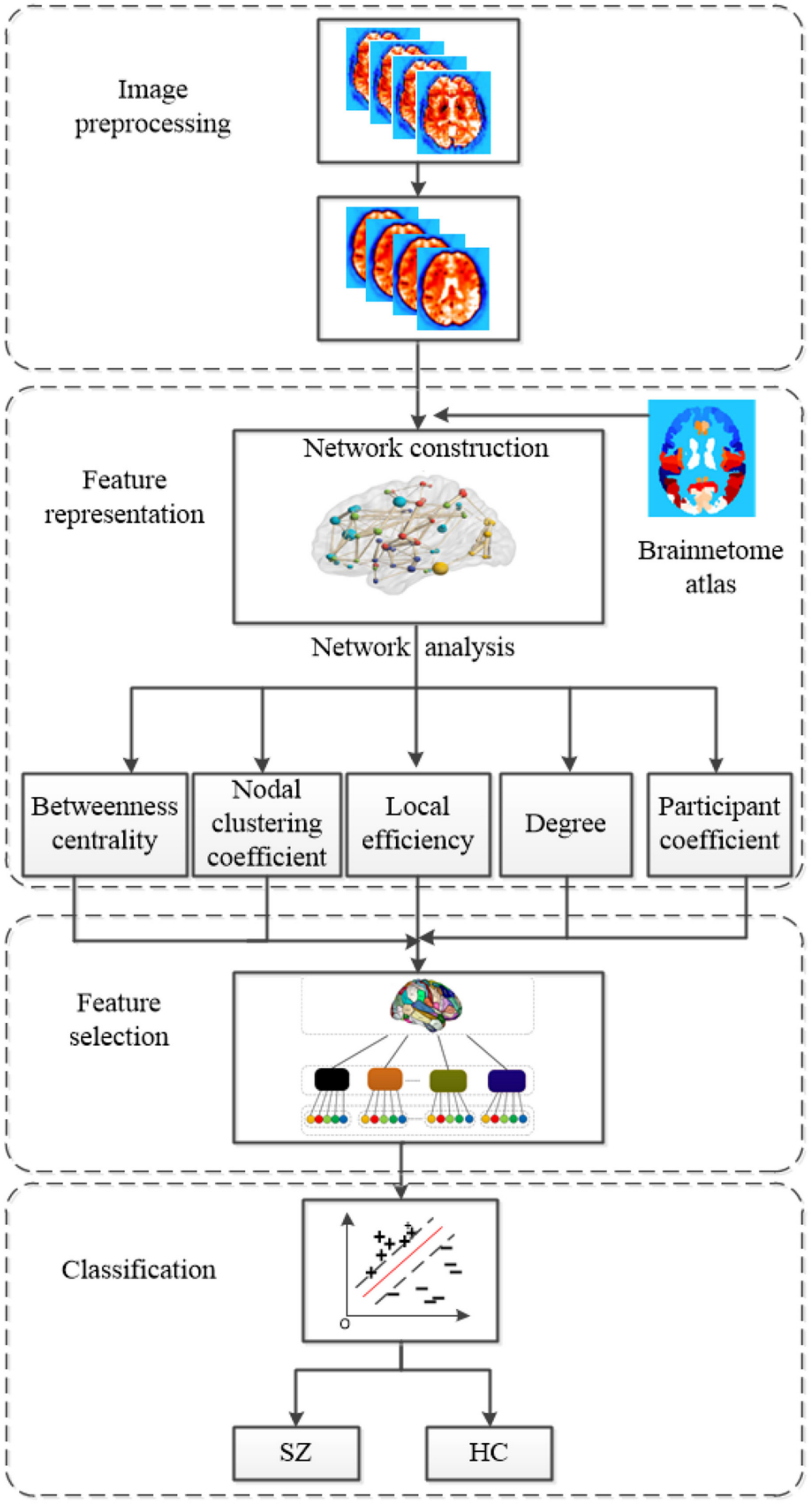
The overall framework of our proposed method using multi-view graph measures of functional brain network for SZ/HC classification.

In this paper, we propose an improved method based on multi-view graph measures to identify SZs from HCs. Functional brain networks are constructed based on fMRI scans. Nodes of functional brain network are brain regions parcellated with the Brainnetome atlas (Fan et al., 2016), and edges of functional brain networks are determined by Pearson's correlation coefficients. Five local graph measures are calculated from functional brain networks by graph theoretical approach as features. The five local graph measures include betweenness centrality, nodal clustering coefficient, local efficiency, degree and participation coefficient. In order to consider the relationship of features within the same region, firstly we need to group graph measures according to brain regions defined by Brainnetome atlas. Then Sparse Group Lasso feature selection method is employed to select the most important regions as well as discriminative features within the selected regions. Finally, support vector machine (SVM) is trained for SZ identification. Our experiments are conducted on 145 samples with fMRI data, including 74 HCs and 71 SZs. Our proposed method achieves a mean classification accuracy of 93.10% using a leave-one-out cross-validation (LOOCV) scheme. The overall framework of our proposed method is shown in Figure 1, which consists of four main components include image preprocessing, feature representation, feature selection, and classification with SVM classifier. The code for this classification framework is available for download at https://github.com/xyzxzj/SZClassification.

## 2. Materials and Methods

### 2.1. Subject Description and Image Preprocessing

The data involved in this study is collected by the Center for Biomedical Research Excellence (COBRE). COBRE^1^ dataset consists of 148 subjects with functional and anatomical MRI data. 74 HCs and 71 SZs of the dataset are employed for our subsequent experiments owing to the class labels of the other three subjects are not given. During the scan, all participants are asked to remain relaxed and keep their eyes open. A brief summary of demographic information of subjects is listed in Table 1.

**Table 1:** Demographic information of 145 subjects from COBRE dataset.

**Type**	**Number**	**Age**	**Gender (M/F)**
SZ	71	38.1 ± 13.9	57/14
HC	74	35.8 ± 11.5	51/23

All of the fMRI images are preprocessed by using Data Processing & Analysis for Brain Imaging (DPABI) (Yan et al., 2016). The preprocessing procedure is as follows: the first 10 volumes of functional runs are removed owing to the fMRI signal instability. Then, the rest volumes are performed slice time correction, head-motion correction, and co-registration of T1-weighted MRI images and fMRI images. After that, the fMRI images are normalized to Montreal Neurological Institute (MNI) space and resampled to 3 × 3 × 3*mm*^3^ voxels. Smooth (4-mm FWHM) and band-pass filter (0.01–0.1Hz) are applied to the images which are transformed to MNI space.

In order to construct time series matrices for all subjects, first all brain images are parcellated into 246 regions by registering images to the Brainnetome atlas after fMRI data preprocessing. Then we extract the averaged time series for each of 246 brain regions for each subject. The time series of each brain region is derived from averaging fMRI signals of all voxels within the region. Finally, a time series matrix consists of 246 regional time series.

### 2.2. Feature Representation

#### 2.2.1. Brain Network Construction

A network is composed of a collection of nodes and links. It can be described as a graph *G* = *(V, E)*, where *V* denotes the set of nodes and E is the set of links. There are four types of network topology, including weighted undirected, weighted directed, binary undirected and binary directed. In this study, the functional connectivity network is represented by an weighted undirected graph. The nodes in functional connectivity network usually are defined by brain regions, and links can represent temporal correlation in activity between pairs of nodes. Given a time series matrix, we can construct a functional connectivity network by calculating Pearson correlation coefficients (Pedersen et al., 2018) between signals of all pairs of regions. The generated functional brain network has 246 × (246 − 1)/2 = 30, 315 weighted edges under the condition of 246 brain regions and the strength of each edge is the Pearson correlation coefficient between a pair of connected nodes.

#### 2.2.2. Brain Network Analysis

A great deal of functional connections in the network may lead to feature redundancy. A threshold *t* is employed in the dense network to keep a certain proportion of edges with the highest correlation. Graph-theoretic measures can quantify topological organization of network. Thus, we can extract some measures which can characterize the global or local functional connectivity from the threshold network. We compute 5 local graph measures using brain network analysis as feature representations, including degree, betweenness centrality, nodal clustering coefficient, local efficiency, and participation coefficient.

Degree is the most fundamental and important measure to characterize the centrality of nodes. In general, nodes with a higher degree are more important in networks. Betweenness centrality can also reflect the centrality of nodes. The betweenness centrality of a brain region can measure its ability on information transmission. Nodal clustering coefficient represents the possibility that any two neighbors of a given node are also neighbors of each other. It measures the ability of the node on functional segregation. Local efficiency measures the efficiency of a subnetwork formed by a given node and all its direct neighbors to transfer information. Local efficiency is related to the shortest path length of the node, the shorter the shortest path length, the greater the local efficiency of the node, the faster the information transmission in the subnetwork. Participation coefficient of a node measures its diversity of intermodular interconnections. The nodes with low participation coefficient but high degree in the module are regarded as provincial hubs, it indicates that the hubs are likely to have a great impact on the modular segregation. These five local measures play an important role in information exchange of functional networks. They can be calculated as follow:

(1) $$\begin{array}{l} K\left(i\right)=\underset{j\in N}{\sum }a_{ij} \end{array}$$

(2) $$\begin{array}{l} B\left(i\right)=\frac{1}{\left(N-1\right)\left(N-1\right)}\underset{m\;\neq \;j\;\neq \;i}{\sum }\frac{n_{mj}\left(i\right)}{n_{mj}} \end{array}$$

(3) $$\begin{array}{l} C\left(i\right)=\frac{2sw_{i}}{\left(K_{i}\left(K_{i}-1\right)\right)} \end{array}$$

(4) $$\begin{array}{l} E_{loc}\left(i\right)=\frac{1}{N_{G_{i}}\left(N_{G_{i}}-1\right)}\underset{j\neq h\neq G_{i}}{\sum }\frac{1}{l_{jh}} \end{array}$$

(5) $$\begin{array}{l} PC\left(i\right)=1-\underset{m\in M}{\sum }{\left(\frac{k_{i}\left(m\right)}{k_{i}}\right)}^{2} \end{array}$$

where *K*(*i*), *B*(*i*), *C*(*i*), *E*_*loc*_(*i*), and *PC*(*i*) are the degree, betweenness centrality, clustering coefficient, local efficiency, and participation coefficient of node *i*, respectively. *N* is the number of nodes in a network, *a*_*ij*_ = 1 if node *i* and node *j* are connected, *a*_*ij*_ = 0 otherwise; *n*_*mj*_(*i*) is the number of shortest paths between *m* and *j* that pass through node *i*, and *n*_*mj*_ is the number of shortest paths between *m* and *j*; *sw*_*i*_ is the sum of the weights of all the connected edges between the neighbors of node *i*; *G*_*i*_ is the subnetwork that contains node *i* and its all direct neighbors, *N*_*G*_i__ is the number of nodes in the subnetwork *G*_*i*_, *l*_*jh*_ is the length of shortest path between node *j* and node *h* in the subgraph; *M* denotes the set of modules, *k*_*i*_ is determined as the number of links between *i* and the nodes within module *m*.

In this study, we adopt the Brain Connectivity Toolbox (http://www.brain-connectivity-toolbox.net) (Rubinov and Sporns, 2010) to calculate these five local graph measures. For each local graph measure (gm), we compute 246 values corresponding to the 246 brain regions. Therefore, the dimension of the final feature vector for each subject is 1,230.

### 2.3. Feature Selection

The raw feature matrices have high dimension, multiple redundancy and multi-noise characteristics. Thus, applying a suitable feature selection algorithm to identify features related to SZ/HC identification and remove unnecessary information appears especially important. Least absolute shrinkage and selection operator (Lasso) (Chan et al., 2015) is widely used in various areas due to the very low data requirements. In addition, lasso can filter variables and reduce the complexity of the model. It aims to select the most important features from dense data matrix by using *l*_1_ norm constraint. The optimization problem can be formulated as follow:

(6) $$\begin{array}{l} \underset{\alpha }{\min}||y-X\alpha |{|}^{2}+{\text{λ}}_{1}||\alpha |{|}_{1} \end{array}$$

where *X* denotes an *n* × *p* feature matrix, and *n* is the number of subjects, *p* represents the dimension of a feature vector. *y* is defined as a class label, α is a coefficient vector, and λ_1_ is a regularization parameter.

Graph measures within the same region usually have a certain correlation. However, Lasso has not consider the relationship between graph measures derived in the same brain region. Hence we use the priori information of brain regions to group measures and then perform feature selection based on this feature grouping structure. Group Lasso (GLasso) (Yuan and Lin, 2006), a group variable selection method, is the extension of Lasso. It can select the most important groups by grouping all the variables and penalizing the *l*_2_ norm of each group. The effect is that we can eliminate the entire set of coefficients into zero at the same time and then this set of features are excluded. The objective function of GLasso is as follow:

(7) $$\begin{array}{l} \underset{\alpha }{\min}||y-X\alpha |{|}^{2}+{\text{λ}}_{2}\overset{M}{\underset{j=1}{\sum }}w_{j}||\alpha_{G_{j}}|{|}_{2} \end{array}$$

where α_*G*_*j*__ denotes the set of coefficients of all features in the group *G*_*j*_, *w*_*j*_ is a weight for group *G*_*j*_.

Actually, there are also many redundant features in the important groups selected by GLasso. It is necessary to perform another feature selection to choose the most important features from these selected groups. Sparse Group Lasso (SGLasso) (Liu et al., 2009) is introduced to select the most significant groups as well as the discriminative features within the selected groups by adding *l*_1_ and *l*_2_ penalties. The objective function of the SGLasso can be written as:

(8) $$\begin{array}{l} \underset{\alpha }{\min}||y-X\alpha |{|}^{2}+{\text{λ}}_{1}||\alpha |{|}_{1}+{\text{λ}}_{2}\overset{M}{\underset{j=1}{\sum }}w_{j}||\alpha_{G_{j}}|{|}_{2} \end{array}$$

**Figure 2:**
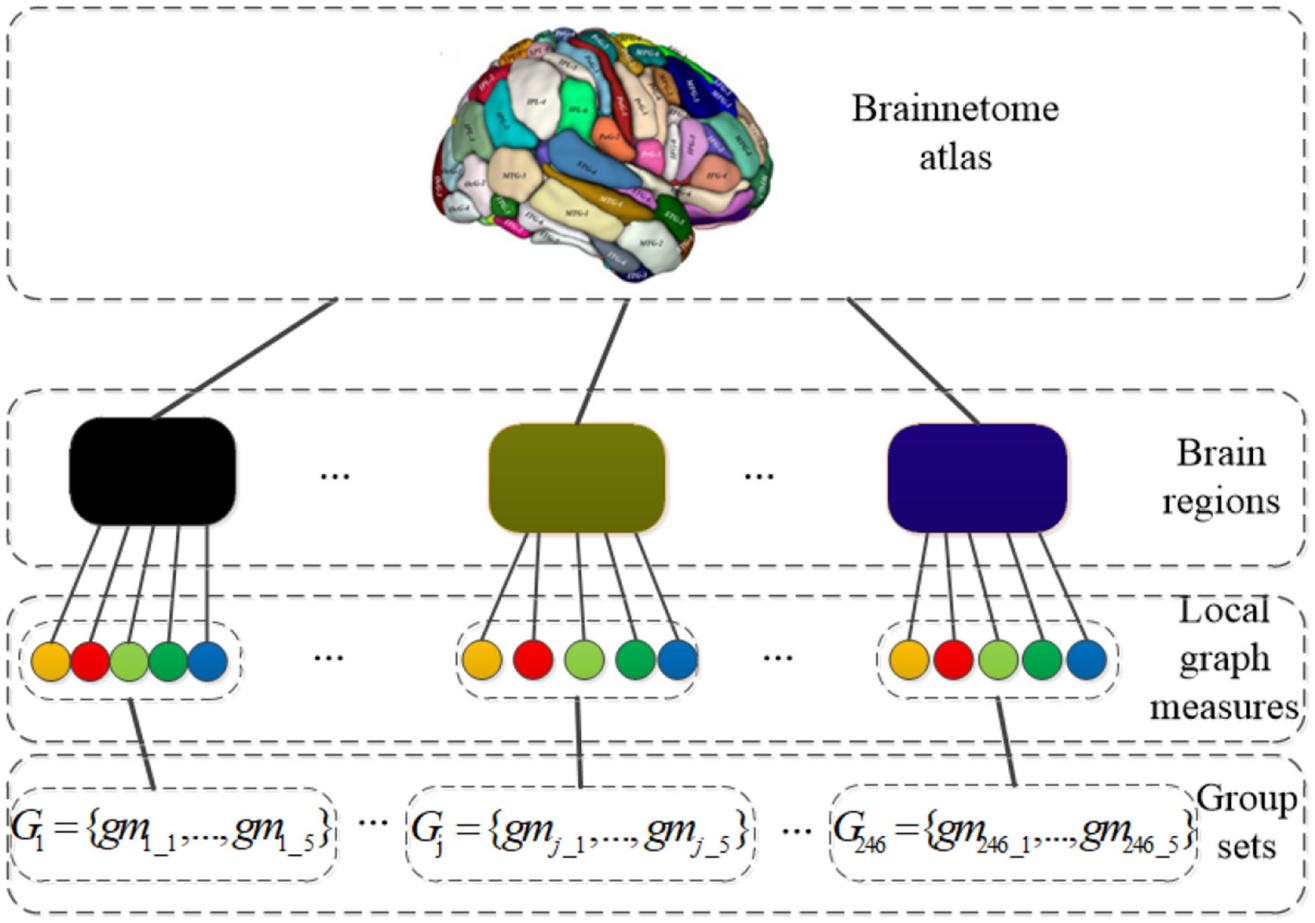
The grouping structure: the nodes in the third layer represent local graph measures and the blocks in the second layer represent brain regions; *G*_*j*_ = {*gm*_*j*_1_, …, *gm*_*j*_5_} is a group set which consists of 5 local graph measures calculated for j_th region.

Before performing SGLasso, 1230-dimensional feature vector for each subject is grouped as *G* = {*G*_1_, …, *G*_*j*_, …, *G*_*M*_} according the brain regions defined by Brainnetome atlas. *M* is the number of groups. *G*_*j*_ = {*gm*_*j*_1_, *gm*_*j*_2_, *gm*_*j*_3_, *gm*_*j*_4_, *gm*_*j*_5_} is a group consists of 5 local graph measures calculated for j_th region. The grouping structure is shown in Figure 2. In addition, z-score transformation is used to normalize the feature matrix before feature selection. It is worth noting that, after feature selection, those features are kept with corresponding regression coefficients greater than the mean value of absolute values of all elements in coefficient vectors.

### 2.4. Classification

SVM (Chang and Lin, 2011) is widely applied in various fields such as natural language processing, target detection, pattern classification due to its good performance as a supervised machine learning approach. The choice of SVM kernel functions is critical to their performance. In this study, we choose the linear kernel SVM (LSVM) to identify SZs from HCs. Linear kernel is mainly used in linear separability cases, and the dimension of the feature space and input space is the same. It performs good classification in most linear separable problems owing to the less parameters and fast calculation. The formulation of SVM model and linear kernel function are as follows:

(9) $$\begin{array}{l} \underset{\lambda }{\max}-\frac{1}{2}\sum_{i=1}^{N}\sum_{j=1}^{N}\lambda_{i}\lambda_{j}y_{i}y_{j}K(x_{i},x_{j})+\sum_{i=1}^{N}\lambda_{i}\\ s.t.\sum_{i=1}^{N}\lambda_{i}y_{i}=0 \end{array}$$

(10) $$\begin{array}{l} 0\leq \lambda_{i}\leq C,i=1,2,...,N\\ \;K(x_{i},x_{j})=<x_{i},x_{j}> \end{array}$$

where λ is the Lagrange multiplier, *N* is the number of samples, *x*_*i*_ represents the feature vector of the i-th sample, and *y*_*i*_ is the label corresponding to *x*_*i*_, *K*(., .) denotes the kernel function, *C* is determined as the soft margin parameter.

After feature selection, the optimal feature set *X* = {*x*_1_, …, *x*_*i*_, …, *x*_*n*_} is used as the input to SVM classifier, *i* = 1,…, *n*. Giving a test subject x, the trained SVM will predict its label based on a decision function P(x) as follows:

(11) $$\begin{array}{l} P\left(x\right)=sign\left(\overset{N}{\underset{i=1}{\sum }}{\text{λ}}_{i}y_{i}K\left(x_{i},x\right)\right) \end{array}$$

## 3. Experiments and Results

### 3.1. Experiment Settings

In our study, the classification performance of our proposed method is estimated by adopting LOOCV scheme. LOOCV scheme is not affected by the random sample partitioning because n samples are only divided into *n* subsets in a unique way, each subset contains one sample. Each subset will be tested as a test data in turn while remaining subjects as the training data. In addition, we usually adopt the LIBSVM library (Chang and Lin, 2011) to solve SVM classification. We further calculate classification accuracy (ACC), sensitivity (SEN), specificity (SPE) to measure the performance of the method. These three metrics can be written as follows:

(12) $$\begin{array}{l} ACC=\frac{TP+TN}{TP+FP+FN+TN} \end{array}$$

(13) $$\begin{array}{l} SPE=\frac{TN}{TN+FP} \end{array}$$

(14) $$\begin{array}{l} SEN=\frac{TP}{TP+FN} \end{array}$$

where true positive (TP), true negative (TN), false negative (FN), and false positive (FP) are defined as the number of correctly classified SZs, HCs and misidentified SZs, HCs, respectively. In addition, the area under receiver operating characteristic (ROC) curve (AUC) is also used to evaluate overall classification performance of our method.

At the stage of feature representation, we set *t* = [0.1, 0.12, …, 0.48, 0.5] to represent a collection of threshold values from 0.1 to 0.5 by the step of 0.02, and then calculate the 5 local graph measures at these 21 thresholds. The two regularization parameters for SGLasso are set as λ_1_ = [1, 2, 3, 4, 5, 6, 7, 8, 9, 10] and λ_2_ = [0.1, 0.2, 0.3, 0.4, 0.5, 0.6, 0.7, 0.8, 0.9, 1.0], which are optimized with the grid search algorithm.

### 3.2. Identification Performance for SZ

**Figure 3:**
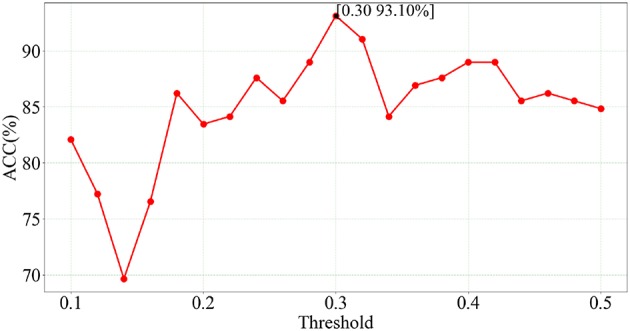
Classification accuracies for SZ identification based on different network thresholds.

We use LSVM to perform SZ/HC classification on the optimal feature set obtained from feature selection of SGLasso at each of 21 thresholds. The classification results corresponding to 21 thresholds are showed in Figure 3.

According to Figure 3, we can see that the best accuracy (93.10%) is achieved at *t* = 0.30. Furthermore, the classification accuracies at these 21 thresholds are all higher than 70%. In addition, the number of selected features is 55 and SEN, SPE, AUC values are 92.96%, 93.24%, 0.950, respectively. The experimental results indicate that the feature combination of five local measures extracted at *t* = 0.30 has a relatively strong correlation with SZ identification.

## 4. Discussion

### 4.1. Comparison With Different Feature Selection Methods

In order to demonstrate the SGLasso method is more effective than the common feature selection methods based on these five local measures for SZ classification, we compare four feature selection methods. The first one is *t*-test which is the one of the most basic feature selection method and the most critical part of this method is selecting features based on the *p*-value (i.e., 0.05). The rest methods are Lasso, GLasso and Elastic Net (Enet). These three methods are based on linear sparse models. GLasso and Enet are the extension of Lasso. GLasso is used to solve *l*_1_/*l*_*q*_-norm regularized problem. Enet is used for the situations where features are related to each other and always produce valid solution.

**Figure 4:**
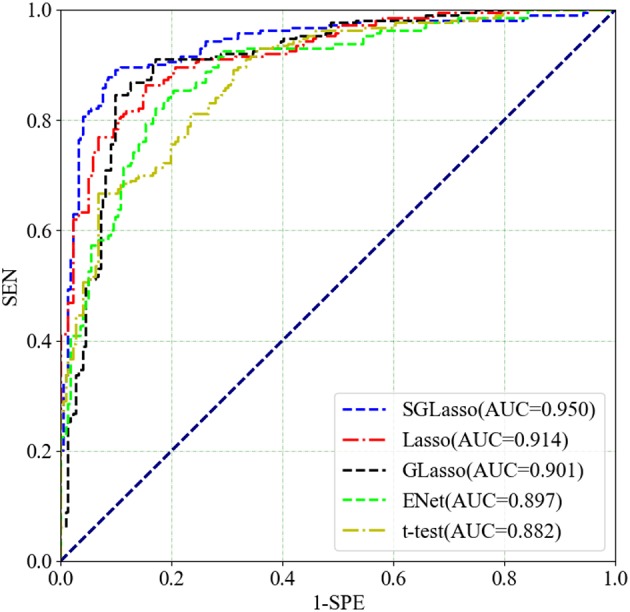
ROC curves for SZ/HC classification for different feature selection methods.

These four feature selection methods perform the same experimental procedure as SGLasso for the sake of fairness. It's worth noting that the five local graph measures are extracted at the threshold of 0.30. Table 2 shows the experimental results of the above mentioned four methods and SGLasso feature selection method. As we can see that SGLasso method selects the least features (55) but achieves the best ACC (93.10%), SEN (92.96%), SPE (93.24%). The ROC curves for SZ/HC classification for different feature selection methods as shown in Figure 4. We notice that SGLasso achieves the highest AUC (0.950) than other four feature selection methods. Experimental result shows that considering within- and between- group sparsity is likely helpful for selecting significant features that are effective for SZ identification.

**Table 2:** Classification with different feature selection methods.

**Methods**	**Number of selected features**	**ACC (%)**	**SEN (%)**	**SPE (%)**
*t*-test	153	78.62	80.28	77.03
Lasso	123	83.45	88.73	78.38
GLasso	225	86.21	85.92	86.49
ENet	64	85.52	84.51	86.19
SGLasso	55	93.10	92.96	93.24

### 4.2. Comparison With Different Classifiers

In order to prove that LSVM is optimal to conduct classification in this context, a series of comparative experiments using several SVMs with different kernels including Radial Basis Function kernel (RBF), Ploynomial kernel (Poly), Sigmoid kernel (Sigm) under the same condition as the LSVM have been performed. These SVMs are denoted as RBF-SVM, Poly-SVM, Sigm-SVM, respectively. The experimental results of SVMs with different kernels are shown in Table 3. It is worth mentioning that bold text indicates that the best result is obtained on a certain evaluation metric.

**Table 3:** Comparison with other SVMs using different kernels.

**Methods**	**ACC (%)**	**SEN (%)**	**SPE (%)**	**AUC**
RBF-SVM	80.00	76.06	83.78	0.8601
Poly-SVM	82.07	77.46	86.49	0.8506
Sigm-SVM	87.59	83.10	91.89	0.9393
LSVM	**93.10**	**92.96**	**93.24**	**0.950**

*Bold text indicates that the best result is obtained on a certain evaluation metric*.

In addition, we also compare four commonly used classifiers, such as k-nearest neighbors (KNN), Random Forest (RForest), NaiveBayes (NBayes), and Linear Discriminant Analysis (LDA). These classifiers are all implemented on the platform of Matlab2016a. We evaluate the performance of the above four classifiers under the same conditions as LSVM. The experimental results of these five classifiers are shown in Table 4. As can be seen from Tables 3, 4, LSVM can achieve the best classification performance than other classifiers.

**Table 4:** Comparison with other commonly used classifiers.

**Methods**	**ACC (%)**	**SEN (%)**	**SPE (%)**	**AUC**
KNN	82.07	74.65	89.19	0.7912
RForest	77.93	74.65	81.08	0.8378
NBayes	84.83	83.10	86.49	0.9069
LDA	90.34	87.32	93.24	0.9418
LSVM	**93.10**	**92.96**	**93.24**	**0.950**

*Bold text indicates that the best result is obtained on a certain evaluation metric*.

### 4.3. Regularization Parameter Selection

The regularization parameters of SGLasso have a great influence on the results of feature selection. Using different regularization parameters, the selected features are also different. It affects not only the feature dimension, but also the final classification performance. Therefore, selecting the appropriate regularization parameters can improve the efficiency of SGLasso method and obtain more effective features associated with the labels.

**Figure 5:**
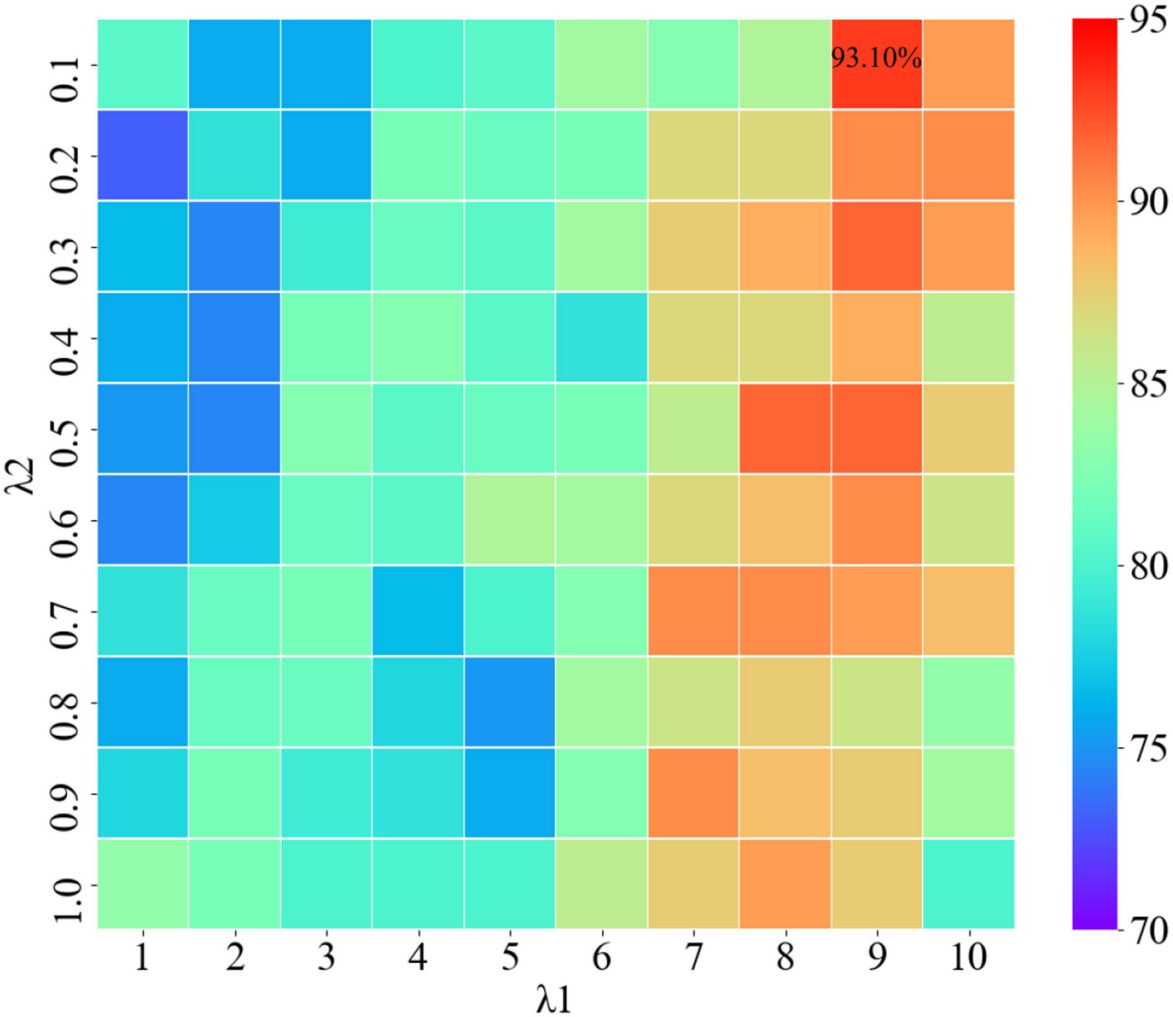
Classification results using different combination of λ_1_,λ_2_.

The two regularization parameters of SGLasso are λ_1_ and λ_2_. λ_1_ is used to control the model sparseness, and λ_2_ can control the sparse constraint of each feature group. We use the grid search algorithm to find the optimal combination of regularization parameters. Figure 5 shows the classification results using different combination of λ_1_, λ_2_. According to Figure 5, when the parameter combination is (λ_1_=9, λ_2_=0.1), the features obtained from SGLasso feature selection method are the most effective for SZ/HC classification.

### 4.4. Regression Coefficient Selection

In general, the non-zero elements in the coefficient vector α generated from the SGLasso feature selection algorithm indicate that the corresponding features are selected. In order to retain the least but most informative features according to α, we test the impact of the three coefficient selection strategies on classification performance. We named these three strategies as SGLasso_absZ, SGLasso_absM, and SGLasso_absMS. The description of these three strategies is as follows:

- SGLasso_absZ is a common strategy to retain non-zero coefficients of α.
- SGLasso_absM strategy is to retain those coefficients which are greater than the mean value of absolute values of all elements in α.
- SGLasso_absMS strategy is more strict for selecting coefficients, since it retains the coefficients which are larger than the mean value of absolute values of all non-zero coefficients in α.

**Figure 6:**
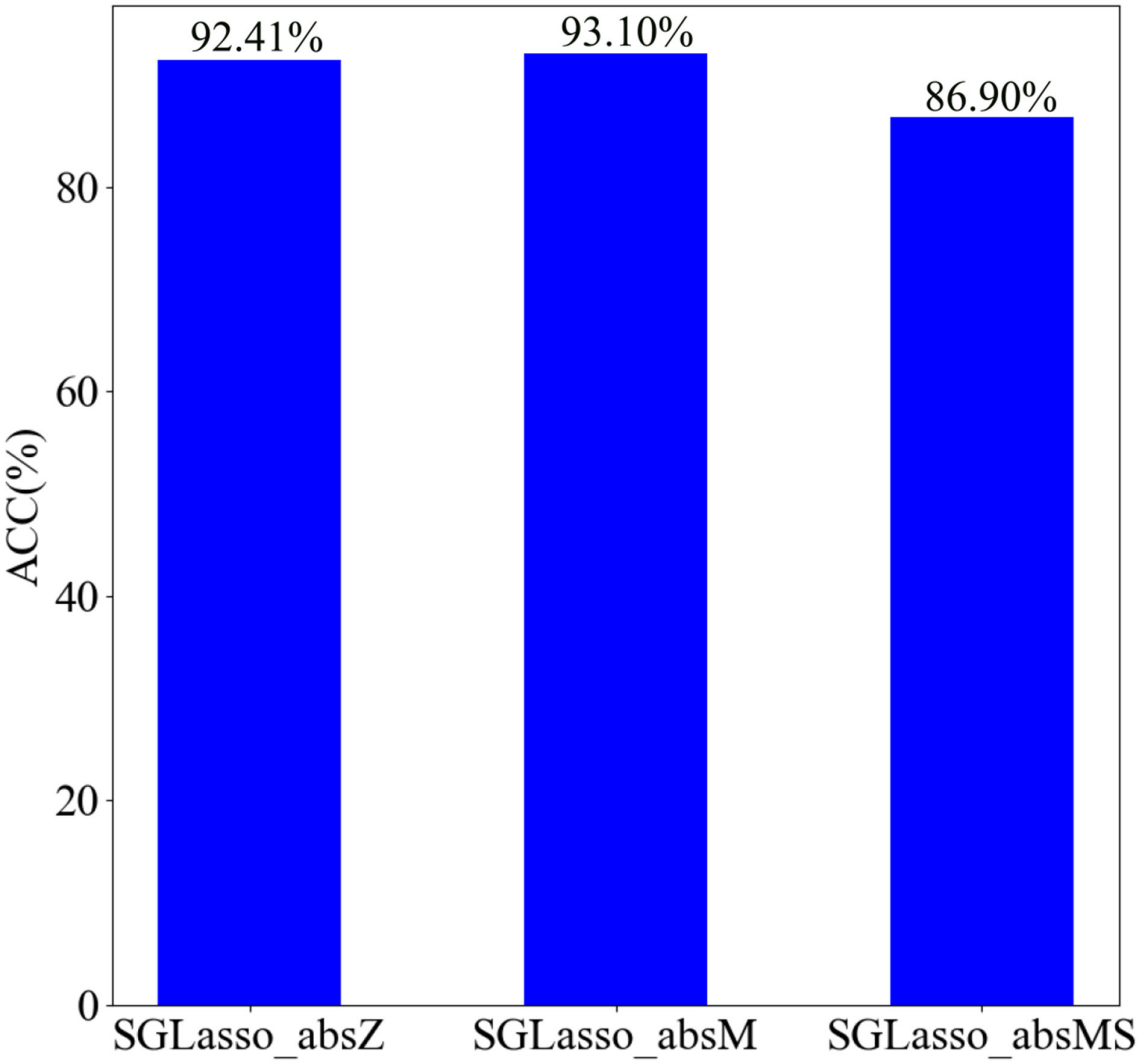
Classification results using three different regression coefficient selection strategies.

We apply the above mentioned three strategies to feature selection, and then select the corresponding features according to the retained coefficients in α. SVM performs SZ identification using these selected features. The classification results using three different regression coefficient selection strategies are shown in Figure 6. According to Figure 6, the classification accuracy is the best when using SGLasso_absM strategy. Experimental result indicates that using SGLasso_absM strategy in feature selection can select the most effective features for SZ/HC classification. Therefore, we finally choose the SGLasso_absM strategy to select the regression coefficients.

### 4.5. Classification Comparison Using Different Feature Combinations

**Figure 7:**
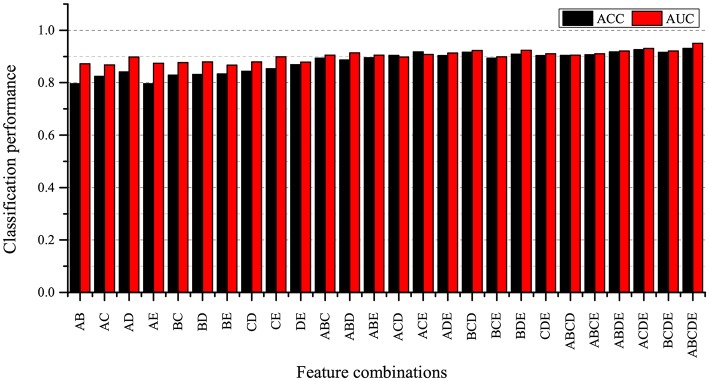
Classification result for different feature combinations. A: betweenness centrality, B: nodal clustering coefficient, C: local efficiency, D: degree, E: participation coefficient.

In order to explore the impact of different feature combinations on SZ/HC identification, we combine these five local measures extracted at the threshold of 0.30 in $C_{5}^{2}+C_{5}^{3}+C_{5}^{4}+C_{5}^{5}=26$ ways. Furthermore, we don't consider individual graph measure because we only investigate multiple measures in this study. We evaluate these 26 feature sets under the same experimental settings. The classification results are shown in Figure 7.

As can be seen from Figure 7, the combination of 5 local graph measures achieves the best classification performance compared to other feature sets. At the same time, we also find that the classification accuracies obtained by using feature sets including two measures are lower than the classification accuracies obtained by using feature sets including three measures, four measures and five measures. It indicates that using fewer measures may not be enough to characterize brain network alternation, and we find that the combination of five local measures can provide more useful information for SZ identification.

### 4.6. Comparison With Existing Classification Methods

To verify the effectiveness of our proposed classification method, we compare some recently proposed methods for SZ classification using fMRI in the literature. Huang et al. (2018) proposed a tree-guided group sparse learning method to select the most important information from FALFF data in four frequency bands and get a classification accuracy of 91.1% by using multi-kernel SVM. Cheng et al. (2015) calculated only betweenness centrality measure to characterize the network. They used the rank of betweenness centrality of all nodes as feature representations and used SVM to classify SZs from HCs.

**Figure 8:**
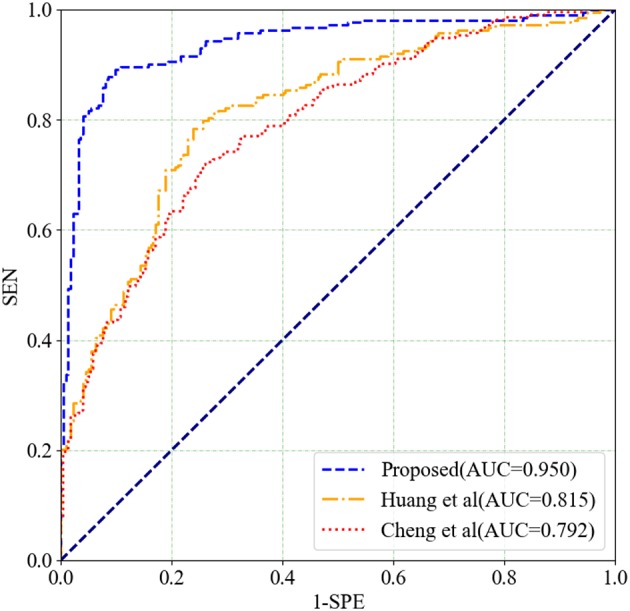
ROC curves for SZ/HC classification for different classification methods.

The two above mentioned methods are performed on the COBRE dataset. The classification results and ROC curves for SZ/HC classification of the two methods and our proposed method are shown in Table 5 and in Figure 8, respectively. According to Table 5 and Figure 8, Our proposed method gets the best ACC (93.10%), SEN (92.96%), SPE (93.24%), and AUC (0.950) values. The experimental result illustrates that our proposed method has made a significant improvement in classification performance on the COBRE dataset.

**Table 5:** Comparison with some existing methods for SZ/HC classification.

**Methods**	**ACC (%)**	**SEN (%)**	**SPE (%)**	**AUC**
Huang et al. (2018)	77.24	77.46	76.58	0.815
Cheng et al. (2015)	74.48	73.53	69.12	0.792
Proposed	**93.10**	**92.96**	**93.24**	**0.950**

*Bold text indicates that the best result is obtained on a certain evaluation metric*.

### 4.7. Analysis of Discriminative Graph Measures and Corresponding Regions

The graph measures selected in the feature selection stage are considered to be related to their corresponding brain regions. Our method can select the most discriminative brain regions as the biomarkers to guide the disease-induced interpretation. There is a total of 145 experiments in the LOOCV scheme due to 145 subjects. And the number of feature occurrence in 145 experiments is introduced to indicate the contribution of the feature to classification. We assume that if the occurrence number of a local graph measure extracted from a certain brain region is greater than 140 in a total of 145 experiments, the brain region is considered to have the most discriminative power to distinguish between SZs and HCs. Based on this hypothesis, 21 salient brain regions have been found. These significant brain regions are shown in Table 6. Five brain regions include left superior frontal gyrus (SFG_L_7_2), right inferior temporal gyrus (ITG_R_7_7), right inferior parietal lobule (IPL_R_6_4), right postcentral gyrus (PoG_R_4_1), and right thalamus (Tha_R_8_7) are related to more than one local graph measure.

**Table 6:** The most discriminative graph measures and corresponding Brainnetome regions.

**Graph measures**	**Hemisphere**	**Brainnetome regions**	**Occurrence number**
Nodal clustering coefficient	SFG_L_7_2	Superior Frontal Gyrus	144
Degree	SFG_L_7_2	Superior Frontal Gyrus	145
Nodal clustering coefficient	SFG_R_7_2	Superior Frontal Gyrus	140
Participation coefficient	SFG_R_7_7	Superior Frontal Gyrus	144
Betweenness centrality	IFG_L_6_3	Inferior Frontal Gyrus	143
Betweenness centrality	OrG_L_6_2	Orbital Gyrus	143
Betweenness centrality	OrG_R_6_6	Orbital Gyrus	145
Betweenness centrality	PrG_L_6_3	Precentral Gyrus	142
Degree	MTG_L_4_4	Middle Temporal Gyrus	145
Betweenness centrality	MTG_L_4_1	Middle Temporal Gyrus	141
Participation coefficient	ITG_R_7_7	Inferior Temporal Gyrus	145
Betweenness centrality	ITG_R_7_7	Inferior Temporal Gyrus	145
Betweenness centrality	FuG_R_3_3	Fusiform Gyrus	145
Betweenness centrality	PhG_L_6_3	Parahippocampal Gyrus	144
Degree	PhG_R_6_5	Parahippocampal Gyrus	145
Local efficiency	IPL_R_6_4	Inferior Parietal Lobule	145
Participation coefficient	IPL_R_6_4	Inferior Parietal Lobule	145
Degree	IPL_R_6_2	Inferior Parietal Lobule	145
Degree	PCun_L_4_3	Precuneus	145
Nodal clustering coefficient	PoG_R_4_1	Postcentral Gyrus	145
Betweenness centrality	PoG_R_4_1	Postcentral Gyrus	145
Local efficiency	PoG_R_4_1	Postcentral Gyrus	143
Degree	PoG_R_4_1	Postcentral Gyrus	145
Participation coefficient	CG_L_7_4	Cingulate Gyrus	145
Betweenness centrality	CG_R_7_3	Cingulate Gyrus	145
Participation coefficient	LOcC_L_4_3	lateral Occipital Cortex	145
Degree	BG_R_6_1	Basal Ganglia	145
Betweenness centrality	BG_R_6_4	Basal Ganglia	145
Participation coefficient	Tha_L_8_8	Thalamus	145
Degree	Tha_L_8_5	Thalamus	145
Degree	Tha_R_8_8	Thalamus	145
Nodal clustering coefficient	Tha_R_8_7	Thalamus	140
Local efficiency	Tha_R_8_7	Thalamus	141

These findings on discriminative brain regions are in agreement with the following studies: superior frontal gyrus, cingulate gyrus, postcentral gyrus (Szeszko et al., 1999; Gur et al., 2000; Arbabshirani et al., 2013; Chyzhyk et al., 2015), parahippocampal gyrus (Shenton et al., 1992; Chyzhyk et al., 2015), middle temporal gyrus, fusiform gyrus and thalamus (Chyzhyk et al., 2015; Li et al., 2019), inferior parietal lobule, inferior temporal gyrus (Peng et al., 1994; Goldstein et al., 1999; Li et al., 2019). However, we cannot report agreement with these regions:inferior frontal gyrus, orbital gyrus, precentral gyrus, precuneus, lateral occipital cortex and basal ganglia.

## 5. Conclusion

In this paper, we propose a method to classify SZs from HCs using multi-view graph measures of functional brain networks. We get five local network measures using graph theoretical approach from multiple views. These measures paly an important role in the information exchange of brain networks. Our proposed method achieves a good classification performance on the COBRE dataset. Experimental results demonstrate that this approach is efficient for the clinical diagnosis of SZ. Furthermore, multiple measures have the potential to be used as clinical biomarkers to differentiate SZs from HCs.

## Data Availability Statement

The imaging data and phenotypic information was collected and shared by the Mind Research Network and the University of New Mexico funded by a National Institute of Health Center of Biomedical Research Excellence (COBRE) grant 1P20RR021938-01A2. The dataset for this study can be found in this website: http://fcon_1000.projects.nitrc.org/indi/retro/cobre.html.

## Author Contributions

JW and JL conceived the project. YX, GT, F-XW, and JL designed the experiments. YX and GT performed the experiments. YX and JL wrote the paper. All authors read and approved the final manuscript.

### Conflict of Interest

The authors declare that the research was conducted in the absence of any commercial or financial relationships that could be construed as a potential conflict of interest.

## Footnotes

^1^http://fcon_1000.projects.nitrc.org/indi/retro/cobre.html
